# Recent advances in the mechanisms of NLRP3 inflammasome activation and its inhibitors

**DOI:** 10.1038/s41419-019-1413-8

**Published:** 2019-02-12

**Authors:** Yang Yang, Huanan Wang, Mohammed Kouadir, Houhui Song, Fushan Shi

**Affiliations:** 1 College of Animal Science and Technology, Key Laboratory of Applied Technology on Green-Eco-Healthy Animal Husbandry of Zhejiang Province, Zhejiang Provincial Engineering Laboratory for Animal Health Inspection and Internet Technology, Zhejiang A&F University, Lin’an, 311300 Zhejiang PR China; 2 Department of Veterinary Medicine, College of Animal Sciences, Zhejiang University, Hangzhou, 310058 Zhejiang PR China; 3 Trustchem Co., Ltd, Nanjing, 210029 Jiangsu PR China

## Abstract

The NLRP3 inflammasome is a multimeric protein complex that initiates an inflammatory form of cell death and triggers the release of proinflammatory cytokines IL-1β and IL-18. The NLRP3 inflammasome has been implicated in a wide range of diseases, including Alzheimer’s disease, Prion diseases, type 2 diabetes, and some infectious diseases. It has been found that a variety of stimuli including danger-associated molecular patterns (DAMPs, such as silica and uric acid crystals) and pathogen-associated molecular patterns (PAMPs) can activate NLRP3 inflammasome, but the specific regulatory mechanisms of NLRP3 inflammasome activation remain unclear. Understanding the mechanisms of NLRP3 activation will enable the development of its specific inhibitors to treat NLRP3-related diseases. In this review, we summarize current understanding of the regulatory mechanisms of NLRP3 inflammasome activation as well as inhibitors that specifically and directly target NLRP3.

## Facts

- The dysfunction of NLRP3 inflammasome activation is implicated in a variety of human diseases.
- The NLRP3 inflammasome can trigger inflammation by sensing a wide range of stimuli, but the specific mechanisms are still unclear.
- Understanding the mechanisms of NLRP3 inflammasome activation will boost the development of its specific inhibitors to treat NLRP3-related diseases.

## Open questions

What factors ultimately determine the NLRP3 inflammasome activation?

Is there a common signaling pathway targeted by NLRP3 inflammasome activation?

Does the specific targeting of NLRP3 itself, and not other components (NEK7, ASC, caspase-1, or IL-1β) or up-/downstream factors of NLRP3 inflammasome produce therapeutic effects?

## Introduction

The innate immunity is the first line of defense that recognizes infection and initiates the process of pathogen clearance and tissue repair. One of the most important complexes which participates in these processes is the inflammasome, first described by Martinon in 2002^1^. The inflammasome is a multi-protein complex that recruits pro-caspase-1 via ASC (the adaptor molecule apoptosis-associated speck-like protein containing a CARD) and then proceeds to cleave the cytokine precursors pro-IL-1β and pro-IL-18 into mature IL-1β and IL-18. Upon activation, the inflammasome also promotes an inflammatory form of cell death named pyroptosis, which is regulated by the N-terminal domain of gasdermin D (GSDMD) by forming pores in the plasma membrane^2–4^.

To date, several inflammasomes have been described, including NLRP3, NLRP1, AIM2, and NLRC4. The NLRP3 inflammasome comprises the sensor molecule NLRP3, the adaptor protein ASC, and pro-caspase-1. The NLRP3 protein contains a pyrin domain (PYD), and the ASC protein harbors PYD and CARD domains. Upon activation, the NLRP3 protein interacts with ASC via PYD, and the CARD domain of ASC recruits the CARD domain of pro-caspase-1 to form NLRP3–ASC–pro-caspase-1 complex, also named NLRP3 inflammasome^5^. The AIM2 (absent in melanoma 2) inflammasome, which senses cytosolic DNA through its C-terminal HIN200 domain, can recruit pro-caspase-1 via ASC to form AIM2–ASC–pro-caspase-1 complex^6^. Unlike NLRP3 and AIM2, the NLRP1 protein contains both PYD and CARD domains, which interact directly with pro-caspase-1 without adaptor protein ASC^7^, but the presence of ASC can enhance NLRP1-mediated caspase-1 activation^7^. NLRC4 contains only a CARD domain, which recruits pro-caspase-1 directly in the absence of ASC to form NLRC4 inflammasome^3^. Infection from pathogenic bacteria, such as *Salmonella*, *Shigella*, and *Pseudomonas aeruginosa*, promotes NLRC4 inflammasome assembly and subsequent pro-caspase-1 cleavage, leading to the secretion of IL-1β and IL-18^8^. Like NLRP1, the presence of ASC in the NLRC4 complex promotes NLRC4-mediated IL-1β and IL-18 release by aggregating ASC specks^3^. Among these inflammasomes, the NLRP3 inflammasome is the most extensively studied.

Activation of NLRP3 inflammasome in macrophages requires two steps: priming and activation. The priming step (signal 1) is provided by inflammatory stimuli such as TLR4 agonists which induce NF-κB-mediated NLRP3 and pro-IL-1β expression, and the activation step (signal 2) is triggered by PAMPs and DAMPs, thereby promoting NLRP3 inflammasome assembly and caspase-1-mediated IL-1β and IL-18 secretion and pyroptosis^9^. However, the priming step is sufficient for human monocytes to mediate caspase-1 activation and IL-1β release^10^. It should be noticed that the priming step is probably not limited to the NF-κB-dependent transcriptional upregulation of NLRP3 and pro-IL-1β^11–14^, as NLRP3 inflammasome can be activated as early as 10 min when treated with signal 1 and signal 2 stimuli simultaneously^13^. Moreover, the post-translational modifications (PTMs) of NLRP3 during the priming step, such as phosphorylation and ubiquitination which we will discuss later, have been suggested to play critical roles in NLRP3 inflammasome activation. In this review, we summarize recent advances in NLRP3 inflammasome activation and inhibitors that specifically and directly target NLRP3 to provide insights into therapeutic strategies for treating NLRP3 inflammasome-mediated diseases.

## Proposed mechanisms of NLRP3 inflammasome activation

### Ion fluxes

**Fig. 1:**
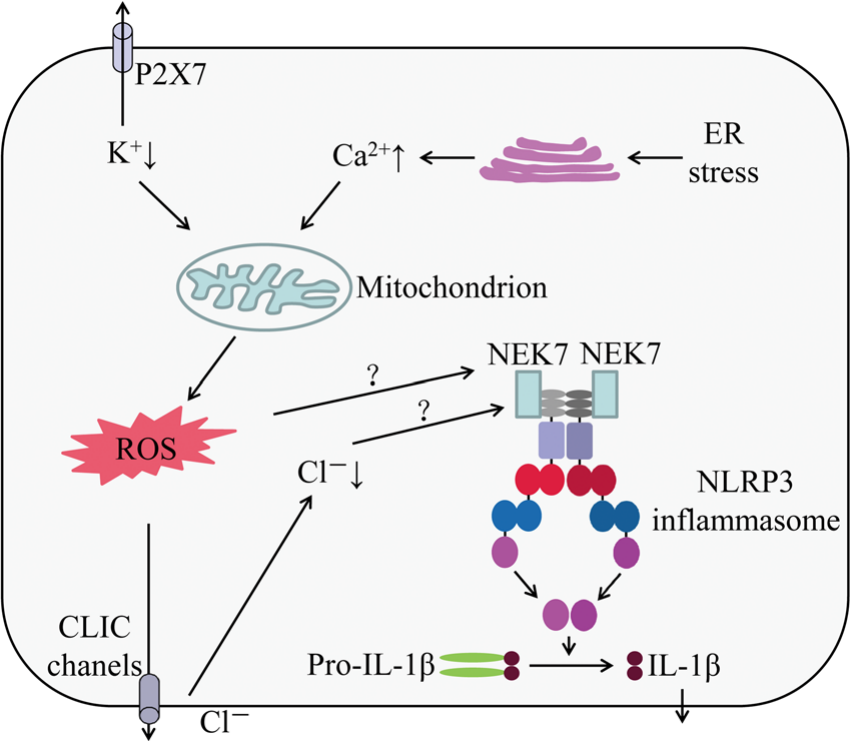
Role of ion fluxes in NLRP3 inflammasome activation. NLRP3 activator-induced K^+^ efflux leads to mitochondrial damage and mtROS production, which can induce enrichment of CLICs in plasma membrane to promote Cl^–^ efflux. CLIC-mediated Cl^–^ efflux can promote NEK7–NLRP3 interaction and subsequent NLRP3 inflammasome assembly. Excessive Ca^2+^ released from ER causes mitochondrial Ca^2+^ overload and mitochondrial damage, leading to mtROS production, which triggers NLRP3 inflammasome activation. The newly identified component of NLRP3 inflammasome NEK7, which can directly bind to NLRP3 protein, also requires K^+^ efflux, ROS production, and Cl^–^ efflux for NLRP3 inflammasome assembly

Ion fluxes, including K^+^ efflux, Ca^2+^ signaling, Na^+^ influx, and chloride efflux, have been identified as critical events in NLRP3 inflammasome activation^15–18^. Due to the diversity of NLRP3 inflammasome activators, it is unlikely that NLRP3 directly interacts with the stimuli. A decrease in intracellular K^+^ concentration was first identified as the common trigger of NLRP3 inflammasome activation. Indeed, numerous NLRP3 inflammasome activators are known to induce K^+^ efflux (including nigericin, ATP, particulate molecules, and crystalline), and intracellular K^+^ decrease is an upstream event in the NLRP3 inflammasome activation^15,19^. A newly identified component of NLRP3 inflammasome NEK7 (NIMA-related kinase 7), which can directly bind to NLRP3 protein, also requires K^+^ efflux for NLRP3 inflammasome assembly (Fig. 1)^20,21^. However, K^+^ efflux is not specific to NLRP3 inflammasome activation. Recent studies have shown that several small molecules, including imiquimod and CL097, can induce ROS production and promote NLRP3 inflammasome activation independent of K^+^ efflux^22,23^. Furthermore, anthrax lethal toxin of *Bacillus anthracis*-induced NLRP1b inflammasome activation also requires intracellular K^+^ decrease for inflammasome activation and IL-1β secretion^19^. Therefore, K^+^ efflux is an important, but not a specific event in the NLRP3 inflammasome activation.

It was reported that Ca^2+^ signaling is required for NLRP3 inflammasome activation. Inhibition of Ca^2+^ mobilization decreases NLRP3 inflammasome activation but has no effect on NLRC4 and AIM2 inflammasomes activation^16,24^. Inositol 1,4,5-trisphosphate (IP_3_), a product of phospholipase C (PLC)-mediated phosphatidylinositol 4,5-bisphosphate hydrolysis, can interact with its receptor IP_3_R on the endoplasmic reticulum and promote Ca^2+^ mobilization and NLRP3 inflammasome activation^16^. However, how Ca^2+^ mobilization induces NLRP3 inflammasome activation is still unclear. It is postulated that excessive ER release of Ca^2+^ causes mitochondrial Ca^2+^ overload and mitochondrial damage leading to mtROS production (Fig. 1), which acts as a central trigger that activates NLRP3 inflammasome^25–27^. On the contrary, other studies indicated that Ca^2+^ signaling is dispensable for NLRP3 inflammasome activation. BAPTA, a strong Ca^2+^ chelator and buffer of cytosolic Ca^2+^, can inhibit NLRP3 inflammasome activation and IL-1β processing independently of its function as a Ca^2+^ chelator^28^. A recent study further revealed that 2-aminoethoxy diphenylborinate (2 APB), a cell-permeable small-molecule inhibitor of Ca^2+^ homeostasis with multiple targets, suppresses NLRP3 inflammasome activation independently of its function as an IP_3_R inhibitor^29^. The principal organelles for Ca^2+^ storage are the ER and the Golgi apparatus, which play important roles both in the maintenance of Ca^2+^ concentration in the resting state and as a source of Ca^2+^ to be released by specific stimuli^30,31^. However, lysosomes also participate in the regulation of Ca^2+^ homeostasis by acting as Ca^2+^ stores that respond to physiological second messengers^32^, and can provide bidirectional communication with the ER Ca^2+^-storage organelle^33^. Thus, further studies are needed to clarify the mechanisms of Ca^2+^ signaling in NLRP3 inflammasome activation.

Na^+^ influx is another important ion involved in NLRP3 inflammasome activation. Schorn et al. reported that MSU crystals stimulation increased sodium ion load and cellular swelling, and the cells were then passively balanced by water influx, which decreased K^+^ below the threshold leading to NLRP3 inflammasome activation^34^. Another study indicated that the influx of extracellular Na^+^ and efflux of intracellular K^+^ are necessary for NLRP3 inflammasome activation in response to stimuli^17^. It seems that Na^+^ influx-induced NLRP3 inflammasome activation is dependent on K^+^ efflux^15^. However, Na^+^ ionophore monensin-induced Na^+^ influx did not lead to NLRP3 inflammasome activation^15^. Therefore, the influx of Na^+^ may not be an absolute requirement for NLRP3 inflammasome activation.

Verhoef et al. was the first to report the role of chloride efflux in NLRP3 inflammasome activation. They showed that decreasing the concentration of extracellular Cl^–^ can induce intracellular Cl^–^ efflux and promote ATP-induced caspase-1 activation and IL-1β production^35^. Since then, studies have demonstrated that Cl^–^ channel inhibitors, including flufenamic acid, IAA94, DIDS, and NPPB, may inhibit NLRP3, but not AIM2 or NLRC4 inflammasome activation^36–38^. The volume-regulated anion channel (VRAC) was initially reported as a critical anion channel in regulating NLRP3 inflammasome activation^36^. The most compelling evidence came from the observation that nonsteroidal anti-inflammatory drugs prevent NLRP3 inflammasome activation by inhibiting chloride efflux through VRAC^37^. Tang et al. further demonstrated that another anion channel, chloride intracellular channel (CLIC), might function as an activator of VRAC^38^. Interestingly, CLIC-dependent chloride efflux was found to be a downstream event of potassium efflux–mitochondrial ROS axis, and CLIC-mediated chloride efflux can promote NEK7–NLRP3 interaction and subsequent ASC oligomerization (Fig. 1)^38^, but the specific mechanism of how the intracellular chloride efflux regulates NEK7–NLRP3 interaction is still unclear. In addition, the relationship between VRAC and CLIC needs further confirmation^18^.

### Reactive oxygen species (ROS) production

ROS generation, especially from the mitochondria, is one of the first identified triggers of NLRP3 inflammasome activation^39–41^. Several studies have shown that most NLRP3 inflammasome agonists can induce mitochondrial ROS generation in different types of cells. For example, fatty acid caused by HFD (high-fat diet) can activate NLRP3 inflammasome in an AMPK–autophagy–ROS-dependent manner^42^. Chemical inhibitors preventing ROS production abrogate numerous stimuli-induced NLRP3 inflammasome activation. Furthermore, multiple agonists that cause cell death and mitochondrial dysfunction increase the oxidation of mitochondrial DNA, which activates NLRP3 inflammasome^43^. However, the mechanisms by which NLRP3 inflammasome senses ROS production are still not well understood. It was suggested that NEK7 rather than NLRP3 itself is a sensor of ROS^20,21^. Imiquimod, a TLR7 agonist, can induce ROS- but not K^+^-dependent NLRP3 inflammasome activation; however, cells deficient in NEK7 failed to produce IL-1β in response to imiquimod stimulation^22^. Other studies also suggest that ROS exerts its role at the priming step, as ROS-specific inhibitors can block NLRP3 inflammasome activation by interfering NLRP3 expression at the priming step, while direct NLRP3 activation is not affected^44^. In contrast, mitochondrial ROS-independent NLRP3 inflammasome activation has also been reported. Jabaut et al. revealed that serum amyloid A-induced mitochondrial ROS-dependent and ROS-independent mechanisms played a role in the NLRP3 inflammasome/IL-1β secretion axis^45^. NLRP3 agonists promote the interaction of NLRP3 with thioredoxin-interacting protein (TXNIP) in a ROS-dependent manner, but caspase-1 activation and IL-1β secretion are not completely inhibited in the absence of TXNIP, indicating that other mechanisms exist^46^. Therefore, additional investigations are needed to elucidate the precise role of ROS in NLRP3 inflammasome activation.

### Lysosomal destabilization

Amyloid β (Aβ), a pathogenic misfolded protein expressed in Alzheimer’s disease, was first identified to activate NLRP3 inflammasome through lysosomal destabilization^47^. Our previous work and other research groups revealed that PrP fibril, another misfolded protein in Prion disease, induced lysosomal destabilization and NLRP3 inflammasome activation^48–50^. Hornung et al. demonstrated that inefficient clearance of large particulate activators (such as silica and alum) leads to lysosomal rupture and cathepsin B release triggering NLRP3 inflammasome activation^51^. In a model of cholesterol crystal-induced NLRP3 inflammasome activation, mice deficient in cathepsin B or L produced few IL-1β compared with wild type^52^. In addition, group B *Streptococcus* and adenovirus type 5-induced NLRP3 inflammasome activation also depends on lysosomal leakage^53,54^. It seems that lysosomal destabilization not only participates in the activation step (signal 2) but also in the priming step (signal 1). In palmitate-induced NLRP3 inflammasome activation, lysosomal calcium signaling regulates the production of pro-IL-1β via stabilization of IL-1β mRNA (signal 1), whereas lysosomal protease cathepsin B contributes to NLRP3 inflammasome activation (signal 2)^55^. This result was further confirmed by a recent study which suggests that multiple cathepsins can promote both pro-IL-1β synthesis and NLRP3 activation^56^. However, it is possible that cathepsin B inhibitors prevent NLRP3 activation through an off-target effect or by targeting other members of the cathepsin family. As reported, CA-074-Me also inhibited anthrax lethal toxin-induced NLRP1b inflammasome activation and caspase-1 cleavage^57^. BMDMs deficient in cathepsin B showed no differences in caspase-1 cleavage and IL-1β secretion upon hemozoin crystals treatment^58^. Muñoz-Planillo et al. reported that the internalization of particulate matter leads to lysosomal membrane damage via phagocytosis, and this damage can trigger NLRP3 inflammasome activation due to K^+^ efflux by opening one or more membrane pores permeable to K^+^. Interestingly, they also found that LPS priming may enhance K^+^ efflux caused by particulate activators, including LL-OMe, AI(OH)_3_, SiO_2_, and CPPD crystals^15^. Therefore, the precise mechanisms of particulate activators-induced lysosomal destabilization in relation to K^+^ efflux need to be fully determined.

### Post-translational modifications of NLRP3

**Fig. 2:**
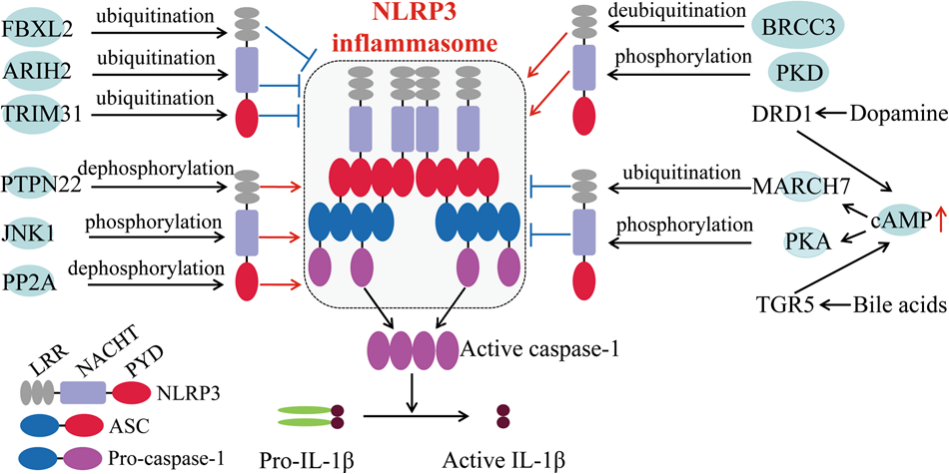
Post-translational modifications (PTMs) regulate NLRP3 inflammasome activation. Schematic structure and PTM sites on different domains of NLRP3 (LRR, NACHT, and PYD). BRCC3 and PTPN22 target the LRR domain of NLRP3 to promote NLRP3 inflammasome activation by deubiquitination and dephosphorylation, respectively. MARCH7 and FBXL2 ubiquitinate NLRP3 LRR domain to inhibit NLRP3 inflammasome activation. Phosphorylation of NLRP3 at S194 and S293 by JNK1 and PKD, respectively, are key events for NLRP3 inflammasome activation. PKA and ARIH2 can phosphorylate and ubiquitinate NLRP3 NACHT domain, respectively, to abrogate NLRP3 inflammasome activation. TRIM31 directly interacts with PYD domain of NLRP3 and promotes its K48-linked ubiquitination, leading to NLRP3 proteasomal degradation and inhibition. Dephosphorylation of NLRP3 PYD domain at S5 by PP2A promotes NLRP3–ASC as well as NLRP3 PYD–PYD interactions

Recent studies indicate that post-translational modifications of NLRP3, including phosphorylation and ubiquitination, play a critical role in NLRP3 inflammasome activation (Fig. 2). Using G5, a small-molecule inhibitor of deubiquitination, Py et al. showed that G5 inhibited NLRP3 inflammasome activation induced by different kinds of activators, including cathepsin-, ROS-, or K^+^ efflux-dependent agonists, but G5 had no effect on AIM2 and NLRC4 inflammasome activation^59^. By screening a deubiquitinating enzyme (DUB) library, they recognized that BRCC3 (BRCA1–BRCA2-containing complex subunit 3) deubiquitinates the LRR domain of NLRP3, which then proceeds NLRP3 activation^59^. Another study suggested that both signal 1 and signal 2 pathways stimulate NLRP3 deubiquitination. The priming step might activate a DUB enzyme targeting a specific domain of NLRP3, whereas ATP signaling might activate another DUB enzyme to deubiquitinate a different domain^13^. The NF-κB inhibitor A20, encoded by TNF-α-induced protein 3 (*TNFAIP3*) genes, prevents spontaneous IL-1β secretion by ubiquitinating the physiological site K133 of pro-IL-1β^60^. A recent study identified the neurotransmitter dopamine (DA) as an endogenous regulator of NLRP3 inflammasome. DA and its receptor DRD1 (dopamine D1 receptor) signaling can suppress neurotoxin-induced neuroinflammation. Mechanistically, DA–DRD1 signaling negatively regulates NLRP3 inflammasome activation through a second messenger cAMP (cyclic adenosine monophosphate), which binds to NLRP3 protein and promotes its ubiquitination and degradation via the E3 ubiquitin ligase MARCH7^61^. Another important E3 ubiquitin ligase involved in NLRP3 inflammasome activation is TRIM31. Following LPS priming, TRIM31 expression is induced as well as NLRP3, and then TRIM31 directly binds to NLRP3 and promotes its K48-linked ubiquitination, leading to NLRP3 proteasomal degradation and inhibition^62^. A recent study indicated that Pellino2 can facilitate NLRP3 inflammasome activation by promoting the K63-linked ubiquitination of NLRP3 as part of the priming phase, and Pellino2 is not involved in TLR-induced NLRP3 and pro-IL-1β upregulation at the priming stage^63^. In contrast, the F-box-only protein 3, FBXO3, participates in LPS-induced NLRP3 protein upregulation during priming^64^. Another F-box protein, F-box L2 (FBXL2), can interact with and ubiquitinate NLRP3 at the Lysine689 residue to promote proteasomal degradation^64^. Thus, TLR stimulation increases the expression of FBXO3, which ubiquitinates and mediates degradation of FBXL2, thereby reducing NLRP3 degradation^64^. Ariadne homolog 2 (ARIH2), another E3 ligase, was reported as a post-translational negative regulator of NLRP3 inflammasome activation in macrophages^65^. ARIH2 can ubiquitinate NLRP3 via the NACHT domain of NLRP3, and the RING2 domain of ARIH2 is required for NLRP3 ubiquitination^65^. Overexpression of ARIH2 promotes NLRP3 ubiquitination and inhibits NLRP3 inflammasome activation^65^.

Ubiquitination of a protein can be regulated by phosphorylation via different approaches, including promotion of recognition by E3 ligase or regulating substrate and ligase interactions^66^. Cholesterol catabolism-derived bile acids can be sensed by the TGR5 receptor (transmembrane G protein-coupled receptor-5), and this interaction increases intracellular cAMP levels and subsequently activates PKA (protein kinase A). PKA catalytic subunit binds to the full length of NLRP3 and phosphorylates its NACHT domain on Ser291. Phosphorylation of Ser291 in turn promotes K48- and K63-linked polyubiquitination and NLRP3 degradation, and hence inhibits NLRP3 inflammasome activation^67,68^. Almost at the same time, Mortimer et al. confirmed the same PKA phosphorylation site Ser295 on human NLRP3 (corresponding to NLRP3 Ser291 in mice)^69^. However, phosphorylation at the same residue may elicit the opposite effect. For instance, Zhang et al. demonstrated that NLRP3 inflammasome stimuli promoted mitochondria-associated membranes (MAMs) localization to the adjacent Golgi membrane and diacylglycerol (DAG) accumulation. DAG accumulation at Golgi activates protein kinase D (PKD), which subsequently phosphorylates NLRP3, resulting in assembly of the fully mature inflammasome^70^. This strongly indicates that the consequences of NLRP3 phosphorylation very likely depend on when and where this modification occurs^70^. Phosphorylation can also occur at the priming step, which is required for NLRP3 transcription. However, recent studies suggest that NLRP3 inflammasome activation can be triggered independently of transcription, indicating the existence of unknown critical regulatory steps in the process^12–14^. Song et al. demonstrated that NLRP3 is phosphorylated during the priming step through the JNK1-mediated NLRP3 phosphorylation at S194, which is a key regulator of NLRP3 inflammasome activation upstream of deubiquitination^71^. Apart from serine phosphorylation, tyrosine phosphorylation of NLRP3 was also reported to be involved in regulating NLRP3 inflammasome activation. PTPN22 (protein tyrosine phosphatase non-receptor 22) interacts with NLRP3 and dephosphorylates it at Tyr861, leading to efficient NLRP3 inflammasome activation and IL-1β secretion, and PTPT22 does not affect AIM2 and NLRC4 inflammasome activation^72^. In addition, phosphorylations at the PYD domain may interrupt the interaction between NLRP3 and ASC. Stutz et al. demonstrated that PP2A (phosphatase 2A) dephosphorylates the Ser5 of NLRP3, which is located at the PYD–PYD interaction interface, and the phosphomimetic residue at this site inhibits NLRP3–ASC, as well as NLRP3 PYD–PYD interaction^73^. Thus, phosphorylation of NLRP3 plays a key role in regulating NLRP3 inflammasome activation, but the difference of phosphorylation likely depends on the timing of priming, stimuli, and the cell type. It will be interesting to investigate other post-translational modifications, such as acetylation, methylation, and succinylation, in the regulation of NLRP3 inflammasome activation.

### Noncanonical inflammasome activation

**Fig. 3:**
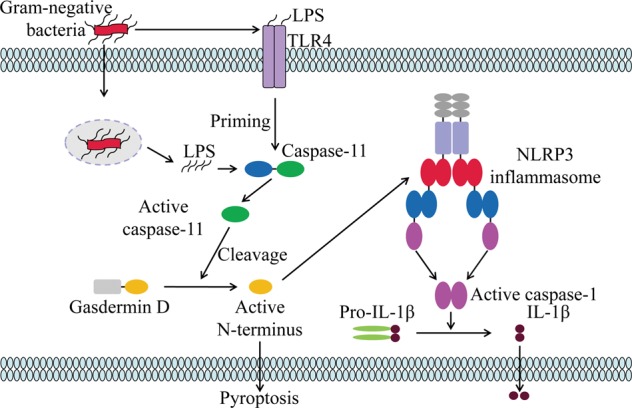
Noncanonical inflammasome activation. LPS of Gram-negative bacteria activates TLR4 to induce caspase-11 transcription. On the other hand, outer membrane vesicles (OMVs) secreted by Gram-negative bacteria act as a vehicle that delivers LPS into the cytosol. LPS that accumulates in the cytosol is directly recognized by the CARD domain of caspase-11, leading to its oligomerization, and then the active caspase-11 cleaves the pore-forming protein gasdermin D within the linker between the *N*-terminal and C-terminal domains. The *N*-terminal domain of gasdermin D can induce pyroptosis and activate NLRP3 inflammasome through a K^+^ efflux-dependent process

The activation pathways of caspase-4/5 in humans and caspase-11 in mice are termed as noncanonical inflammasome activation. Both the canonical and noncanonical inflammasome activation modes eventually lead to cell lysis and the release of proinflammatory cytokines, but their mechanisms differ significantly. Unlike canonical NLRP3 inflammasome activation, noncanonical inflammasome activation is triggered by caspase-11 in mice and caspase-4/5 in humans^4,74^. However, a priming signal to induce caspase-11 transcription is also essential for noncanonical inflammasome activation, due to the low expression levels of caspase-11 in resting cells. In humans, caspase-4 is constitutively expressed in many nonmonocytic cells and monocytes; therefore, cytosolic LPS can activate the noncanonical inflammasome without the priming step^75^. The noncanonical inflammasome can sense a number of Gram-negative bacteria, but not Gram-positive bacteria, which indicates that the outer membrane LPS of Gram-negative bacteria may be the critical activator of the noncanonical inflammasome. Further studies demonstrated that the conserved region lipid A of LPS is responsible for noncanonical inflammasome activation^75–77^. Intracellular LPS or lipid A is directly recognized by the CARD domain of caspase-4/5/11 leading to its oligomerization, followed by the cleavage of the pore-forming protein gasdermin D within the linker between the *N*-terminal and *C*-terminal domains by active caspases^74,78^. The unleashed N-terminal domain of GSDMD targets the plasma membrane and forms membrane pores with an inner diameter of 10–14 nm, which facilitates potassium efflux, pyroptosis, and subsequent NLRP3 inflammasome activation (Fig. 3)^79,80^. Therefore, caspase-4/5/11 do not cleave interleukins but only lead to pyroptosis, and the subsequent potassium efflux-induced NLRP3 inflammasome activation is responsible for caspase-1 activation and IL-1β secretion^75,81^. This evidence points to the interactions between the canonical and noncanonical inflammasomes^82,83^.

### Alternative inflammasome activation

It is well established that NLRP3 inflammasome activation triggers caspase-1 activation and IL-1β maturation through a two-step mechanism. However, compelling evidence shows that LPS alone is sufficient to induce caspase-1-dependent IL-1β maturation and production in human monocytes^10^. Gaidt et al. was the first to identify a new type of inflammasome activation and named it as alternative inflamamsome activation, which is induced by TLR4 signaling pathway without the involvement of other second activators^84^. In alternative activation, NLRP3 is the main factor and its activation requires TRIF (the adaptor protein TIR domain-containing adapter molecule 1) and subsequent cleavage of caspase-8^79^. The major differences between alternative and classical inflammasome activation (canonical and noncanonical) include the non-dependency on K^+^ efflux, the absence of pyroptosome formation, and pyroptosis. In addition, the alternative inflammasome activation is species-specific, as it is restricted to human and porcine monocytes but not observed in murine cells^84,85^. The signaling pathway relies on TLR4–TRIF–RIPK1–FADD–CASP8 to promote NLRP3 inflammasome activation. However, this signaling axis is limited to an alternative inflammasome and has no role in classical NLRP3 inflammasome activation^84^, and the exact mechanism by which caspase-8 activates NLRP3 during alternative inflammasome activation remains unknown^86^. It is speculated that caspase-8-mediated activation of an unknown, intermediate protein is necessary for alternative inflammasome activation^84,86^. Therefore, further studies are needed to identify the missing link between caspase-8 and NLRP3 during alternative inflammasome activation.

### The new synthesis of mitochondrial DNA (mtDNA)

As discussed above, given the diversity of NLRP3 inflammasome agonists, it appears likely that NLRP3 may sense a common triggering pathway induced by intracellular processes. The latest study by Zhenyu Zhong et al. suggests that the generation of oxidized mtDNA (ox-mtDNA) may be the “ultimate” ligand for NLRP3 inflammasome activation^87^. It is already known that mitochondrial damage leads to ox-mtDNA production and is a critical event in NLRP3 activation, but mitochondrial damage cannot trigger NLRP3 activation without priming^88^. Zhong et al. demonstrated that how mitochondria link the priming and activation stages during NLRP3 triggering^87^. LPS priming can increase mtDNA synthesis in mitochondria, which is dependent on the TLR adaptor MYD88 (at early time points), TRIF (at later time points), and IRF1 downstream of both adaptors^87,89^. The newly synthesized mtDNA is responsible for the production of ox-mtDNA, which co-localizes with ASC upon NLRP3 stimulation. They further identified the mitochondrial deoxyribonucleotide kinase UMP-CMPK2 (CMPK2) as the downstream target of IRF1, and CMPK2 as the rate-limiting enzyme controlling the supply of dNTP precursors for LPS-induced mtDNA synthesis^87^. Therefore, the authors identified the critical role of TLR–MyD88/TRIF–IRF1–CMPK2 axis in NLRP3 inflammasome activation and provided new strategies that may be exploited for the treatment of NLRP3-dependent diseases.

### Potential inhibitors of NLRP3 inflammasome activation

**Fig. 4:**
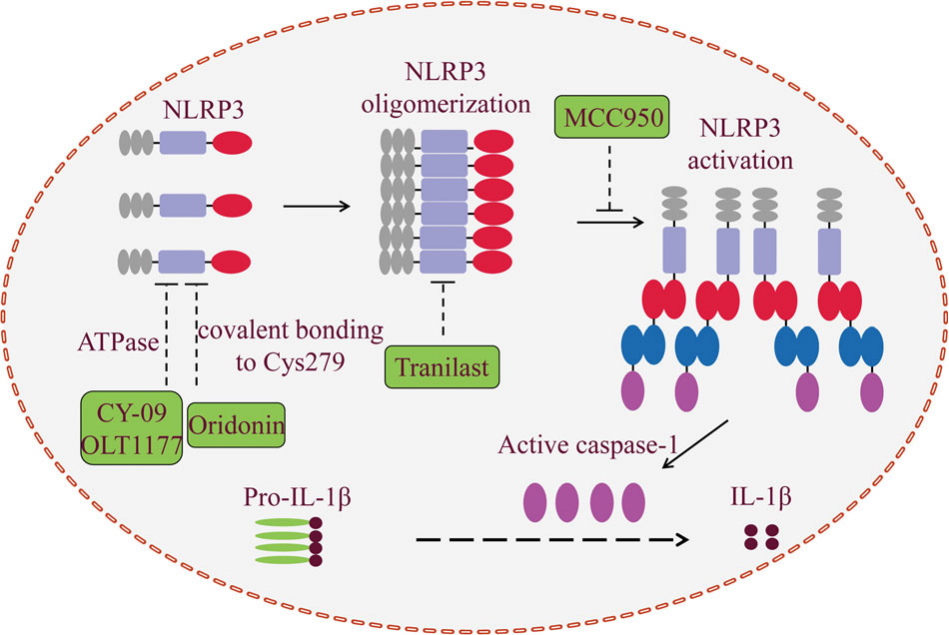
Schematic illustration of the mechanism of NLRP3 inhibitors tested via in vitro and in vivo experimental models. Several molecules have shown inhibitory effects on NLRP3 activation, few of which have been validated in animal models. CY-09 and OLT1177 can inhibit the ATPase activity of the NACHT domain of NLRP3, which is critical for NLRP3 oligomerization. MCC950 is a compound that specifically inhibits NLRP3 inflammasome activation, but its molecular mechanism has not been fully elucidated. Tranilast directly binds to the NACHT domain of NLRP3 to inhibit NLRP3–NLRP3 interaction and the subsequent ASC oligomerization. Oridonin binds to cysteine 279 of NACHT via covalent bond formation to prevent NEK7–NLRP3 interaction and the subsequent NLRP3 inflammasome activation

Involvement of the NLRP3 inflammasome in different kinds of diseases provides new avenues to design drugs targeting NLRP3 inflammasome. To date, clinical treatment of NLRP3-related diseases targets IL-1β with IL-1β antibodies or recombinant IL-1β receptor antagonist, such as canakinumab and anakinra, respectively. In addition, a few small-molecule compounds have shown anti-inflammatory effects on NLRP3 inflammasome activation in vitro, including MCC950^90^, β-hydroxybutyrate (BHB)^91^, Bay 11-7082^92^, dimethyl sulfoxide (DMSO)^93^, and type I interferon^94^. However, most of these inhibitors are relatively nonspecific and have low efficacy. For inhibitors targeting IL-1β, it should be noted that IL-1β secretion is not the only product of NLRP3 inflammasome activation; instead, other proinflammatory cytokines, including HMGB1 and IL-18 may participate in the pathogenesis of these diseases^95,96^. Moreover, IL-1β can be produced by inflammasome-independent pathways or other inflammasomes. Therefore, inhibitors targeting IL-1β may lead to unintended immunosuppressive effects besides preventing NLRP3 inflammasome activation itself. Pharmacological inhibitors specific to NLRP3 inflammasome may be the best choice for treatment of NLRP3-related diseases. Here, we discuss five recently identified pharmacological inhibitors of NLRP3 inflammasome activation and their therapeutic potentials (Table 1). Figure 4 shows the characteristics and the proposed sites of action of the five compounds based on the evidence from in vivo and in vitro experiments.

**Table 1:** Specific inhibitory compounds of NLRP3 inflammasome activation

Agents	Host	Targets	References
MCC950	C57BL6/BMDMs PD model	ASC oligomerization	^90, 99^
CY-09	C57BL6J/BMDMs	NLRP3 ATPase	^98^
OLT1177	C57BL6J/J774A.1 cells, U937 cells	NLRP3 ATPase	^104^
Tranilast	C57BL6J/BMDMs	NLRP3 oligomerization	^109^
Oridonin	C57BL6J/BMDMs	Cysteine 279 of NLRP3	^119^

### MCC950

A small-molecule inhibitor of the NLRP3 inflammasome termed MCC950 was described by Coll et al. in 2015^90^. MCC950 specifically inhibits both canonical and noncanonical NLRP3 inflammasome activation and IL-1β secretion by preventing NLRP3-induced ASC oligomerization in human and mouse macrophages. In contrast, it has no effect on the activation of AIM2, NLRC4, or NLRP1 inflammasome^37,90,97^. In vivo experiments showed that MCC950 reduces IL-1β and IL-18 secretion, thereby alleviating the severity of EAE (experimental autoimmune encephalomyelitis), based on a mouse model of human multiple sclerosis^90^. Mechanistically, MCC950 treatment does not inhibit TLR signaling or the priming step of NLRP3 activation and its inhibitory effect is independent of K^+^ efflux, Ca^2+^ flux, or NLRP3–ASC interactions^90^. Furthermore, MCC950 does not directly inhibit NEK7–NLRP3 or NLRP3–NLRP3 interaction^90^. Thus, it was speculated that MCC950 may bind to NLRP3 and regulate a key step in its activation, such as post-translational modifications^63^. A recent study by Jiang et al. demonstrated that MCC950 can block nigericin-induced NLRP3 inflammasome activation via inhibiting chloride efflux, which acts as the upstream signaling pathway in NLRP3 inflammasome activation^98^. However, it should be noted that nigericin does not lead to chloride efflux, and another NLRP3 inflammasome activation factor ionophore, gramicidin, can trigger chloride influx^15^. More explorations are needed to define this signaling pathway. Gordon et al. recently demonstrated that pharmacological inhibition of NLRP3 inflammasome activation with oral MCC950 treatment could protect against dopaminergic degeneration in a mouse model of PD. These results indicate that MCC950 may represent a promising agent to alleviate dopaminergic degeneration in PD^99^. Thus, the specific inhibitory effect of MCC950 opens the possibility of treating conditions in which either canonical and/or noncanonical NLRP3 inflammasome are involved.

### CY-09

A recent study suggested that the ATPase activity of NLRP3 may be a potential drug candidate for the treatment of NLRP3-related diseases. Jiang et al. found that the cystic fibrosis transmembrane conductance regulator (CFTR) channel inhibitor-C172 has significant anti-inflammatory effects on NLRP3 inflammasome activation^98^. A C172, CY-09, specifically inhibits NLRP3 inflammasome activation, and its inhibitory effect is independent of the signal 1 (NLRP3 and pro-IL-1β expression) and the post-translational modifications step (NLRP3 ubiquitination). Jiang and colleagues confirmed that CY-09 can directly bind to the Walker A motif of NLRP3, but not NLRC4, NLRP1, NOD2, or RIG-1, to abrogate the ATP binding of NLRP3 to inhibit its ATPase activity^98^. This is consistent with a previous study which showed that ATPase activity of NLRP3 is critical for the oligomerization of NLRP3 and its activation^100^. Importantly, CY-09 displayed a good pharmacokinetic profile in terms of safety, stability, and oral bioavailability, and it can be used for blocking NLRP3 inflammasome activation as found in a mouse model of NLRP3-related diseases^98^. Therefore, CY-09 is the first compound recognized to specifically inhibit NLRP3 inflammasome both in vitro and in vivo, and its inhibitory mechanism has been clearly elucidated. Although additional studies are needed to narrow down its effect on other inflammasomes, CY-09 provides a new approach for inhibiting NLRP3 inflammasome activation.

### OLT1177

OLT1177, an active β-sulfonyl nitrile, was initially identified as a candidate for the topical treatment of degenerative arthritis, which has successfully passed phase I clinical trial, and now is being studied at phase II clinical trial for the treatment of acute gouty arthritis^101^. Marchetti et al. showed that OLT1177 could decrease neutrophil infiltration and joint swelling, suppressing proinflammatory IL-1β and IL-6 secretion in the mouse model of zymosan- and monosodium urate-induced arthritis^102^. They further described the anti-inflammatory effect of OLT1177 on NLRP3 inflammasome activation and related diseases^103^. OLT1177 specifically inhibited both canonical and noncanonical NLRP3 inflammasome activation in vitro, and showed no effect on the AIM2 and NLRC4 inflammasomes. OLT1177 reduced caspase-1 activity and IL-1β production in monocytes from patients with CAPS and alleviated the severity of LPS-induced systemic inflammation in vivo. Importantly, no biochemical or hematological adverse effects were observed in humans receiving a high concentration of OLT1177 for 8 days^104^. Like CY-09, OLT1177 exerts its anti-inflammatory effect on NLRP3 inflammasome activation independently of signal 1 (NLRP3 and pro-IL-1β expression) or K^+^ efflux, but directly binds to NLRP3 and inhibits ATPase activity^104^. Thus, OLT1177 specifically prevents NLRP3 inflammasome formation and has the potential to treat NLRP3-related diseases, especially acute gout flares. Future works are needed to investigate the effect of OLT1177 on other inflammasome activation, such as NLRP1, NLRP12, and pyrin inflammasomes.

### Tranilast

To date, dozens of small molecules have been identified as NLPR3 inflammasome inhibitors, and several of them have been studied in mouse models of human diseases. But none of them was thought to be of clinical value until a new study identified the anti-allergic drug Tranilast (N-[3′,4′-dimethoxycinnamoyl]-anthranilic acid). Tranilast, the analog of a tryptophan metabolite, was initially recognized as an anti-allergic agent and used to treat a variety of inflammatory diseases^105^. In addition, Tranilast is a relatively safe drug and it is well accepted by most patients even at doses of up to 600 mg/day for several months^106,107^. It is well known that Tranilast has anti-inflammatory effects, and it can prevent IgE-induced histamine secretion from mast cells^107,108^, but the molecular mechanisms are not clearly elucidated. Huang and colleagues identified Tranilast as a specific NLRP3 inflammasome inhibitor^109^. Like the three specific inhibitors mentioned above, Tranilast does not interfere with the upstream signaling pathways of NLRP3 inflammasome, including NLRP3 and pro-IL-1β expression, K^+^ efflux, mitochondrial damage, ROS production, and chloride efflux, and cannot prevent the newly identified NLRP3 inflammasome component NEK7 from interacting with NLRP3^109^. Tranilast directly binds to the NACHT domain of NLRP3 to inhibit NLRP3–NLRP3 interaction and subsequent ASC oligomerization. Unlike the CY-09 identified by the same group, Tranilast inhibits NLRP3 inflammasome activation via an ATPase-independent manner, by blocking direct NLRP3–NLRP3 interaction^109^. In vivo experiments further showed that Tranilast has significant therapeutic and preventive effects on the mouse models of NLRP3-related diseases. Considering its safety and specific effect on NLRP3 inflammasome, this study provides a potentially practical pharmacological avenue for treating NLRP3 inflammasome-related diseases.

### Oridonin

Oridonin is the major bioactive constituent of *Rabdosia Rubescens*, a widely used over-the-counter (OTC) herbal medicine for the treatment of inflammatory diseases. Oridonin has been reported to exhibit antitumor, anti-inflammatory, and pro-apoptotic effects^110–112^. Previous studies demonstrated that oridonin may suppress MAPK or NF-κB activation and inhibit inflammasome-independent proinflammatory cytokines release, such as TNF-α and IL-6^113–115^. Moreover, oridonin displayed potent therapeutic effects on sepsis, colitis, and neuroinflammation^116–118^. A recent study by He et al. clarified the underlying mechanisms of oridonin in anti-inflammatory activity. Oridonin can specifically inhibit NLRP3 inflammasome activation but has no effect on AIM2 or NLRC4 inflammasome activation, LPS-induced NLRP3, pro-IL-1β expression, and TNF-α production^119^. Oridonin directly binds to the NACHT domain of NLRP3, and the cysteine 279 on NACHT is a covalent binding site of oridonin^119^. Thus, oridonin binds to cysteine 279 of NACHT via a covalent bond to prevent NEK7–NLRP3 interaction and the subsequent NLRP3 inflammasome activation. Importantly, oridonin exerts therapeutic effects on peritonitis, gouty arthritis, and type 2 diabetes in a NLRP3 inflammasome-dependent manner^119^.

## Conclusions

Given the large number and diversity of NLRP3 inflammasome activators, it appears likely that NLRP3 may sense a common triggering pathway induced by intracellular events, but not directly interact with all its agonists. Understanding the mechanism of NLRP3 inflammasome activation and regulation will be critical for developing treatments of NLRP3 inflammasome-related inflammatory diseases. Zhijian James Chen’s very recent study revealed the common triggering pathway for NRLP3 inflammasome activation^120^. Their results showed that diverse NLRP3 stimuli lead to the disassembly of the *trans*-Golgi network (TGN). NLRP3 is recruited to the dispersed TGN (dTGN) via its polybasic 4-lysine motif by binding to the phosphatidylinositol-4-phosphate (Ptdlns4P) on the dTGN^120^. Therefore, the newly identified polybasic region of NLRP3 provides a new strategy for developing therapeutics for the treatment of NLRP3 inflammasome-related inflammatory diseases.

Based on accumulating evidence from recent investigations, a few of the NLRP3 inflammasome inhibitors have been identified and validated via in vitro and in vivo animal models of NLRP3-driven diseases. Among them, the five inhibitors mentioned above displayed the good therapeutic properties, as they directly target NLRP3 itself, but not other components (NEK7, ASC, caspase-1, or IL-1β) up-/downstream of NLRP3 inflammasome activation. Furthermore, the five inhibitors are being used in clinical practice or are being investigated at phase II clinical trials having shown relatively high safety. In conclusion, the search for a full range of NLRP3 inflammasome inhibitors that can efficiently treat NLRP3 inflammasome-related inflammatory diseases is at its foundational stage, and identification of inhibitory agents that are specific to NLRP3 itself is expected to provide the most potent therapeutic strategies.
